# A Phase I Study of the Pan-Notch Inhibitor CB-103 for Patients with Advanced Adenoid Cystic Carcinoma and Other Tumors

**DOI:** 10.1158/2767-9764.CRC-23-0333

**Published:** 2023-09-14

**Authors:** Glenn J. Hanna, Anastasios Stathis, Elena Lopez-Miranda, Fabricio Racca, Doris Quon, Serge Leyvraz, Dagmar Hess, Bhumsuk Keam, Jordi Rodon, Myung-Ju Ahn, Hye Ryun Kim, Andreas Schneeweiss, Josep-Maria Ribera, Daniel DeAngelo, Jose Manuel Perez Garcia, Javier Cortes, Oliver Schönborn-Kellenberger, Dirk Weber, Pavel Pisa, Michael Bauer, Laura Beni, Maria Bobadilla, Raj Lehal, Michele Vigolo, Florian D. Vogl, Elena Garralda

**Affiliations:** 1 Department of Medical Oncology, Center for Head and Neck Oncology, Dana-Farber Cancer Institute, Boston, Massachusetts.; 2 Oncology Institute of Southern Switzerland, EOC, Bellinzona, Switzerland.; 3 Faculty of Biomedical Sciences, Università della Svizzera Italiana (USI), Lugano, Switzerland.; 4 Medical Oncology, Hospital Universitario Ramón y Cajal, Madrid, Spain.; 5 IOB – Institute of Oncology Barcelona and Madrid, Hospital Quironsalud-Barcelona, Barcelona, Spain.; 6 Sarcoma Oncology Research Center, Santa Monica, California.; 7 Charité Comprehensive Cancer Center, Charité Campus Benjamin Franklin, Berlin, Germany.; 8 Department of Medical Oncology, Kantonsspital St Gallen, St Gallen, Switzerland.; 9 Department of Internal Medicine, Seoul National University Hospital, Seoul, Republic of South Korea.; 10 Department of Investigational Cancer Therapeutics, The University of Texas MD Anderson Cancer Center, Houston, Texas.; 11 Samsung Medical Center Sungkyunkwan University School of Medicine, Seoul, Republic of South Korea.; 12 Severance Hospital – Yonsei Cancer Center, Seoul, Republic of South Korea.; 13 National Center for Tumor Diseases (NCT), University Hospital Heidelberg and German Cancer Research Center, Heidelberg, Germany.; 14 Institut Català d'Oncologia (Catalan Institute of Oncology [ICO]), Josep Carreras Research Institute, Barcelona, Spain.; 15 Division of Leukemia, Dana-Farber Cancer Institute, Boston, Massachusetts.; 16 International Breast Cancer Center (IBCC), Pangaea Oncology, Quiron Hospital, Barcelona, Spain.; 17 Medica Scientia Innovation Research, Barcelona, Spain.; 18 Medica Scientia Innovation Research, Ridgewood, New Jersey.; 19 Cogitars GmbH, Heidelberg, Germany.; 20 Cellestia Biotech AG, Basel, Switzerland.; 21 piMedConsulting Ltd, Gersau, Switzerland.; 22 R&D, Cellestia Biotech AG, Epalinges, Switzerland.; 23 Early Drug Development Unit, Clinical Research Program, Vall d'Hebron University Hospital and Institute of Oncology (VHIO) and Medical Oncology, Vall d'Hebron University Hospital (HUVH), Barcelona, Spain.

## Abstract

**Purpose:**

CB-103 selectively inhibits the CSL–NICD (Notch intracellular domain) interaction leading to transcriptional downregulation of oncogenic Notch pathway activation. This dose-escalation/expansion study aimed to determine safety, pharmacokinetics, and preliminary antitumor activity.

**Experimental Design:**

Patients ≥18 years of age with selected advanced solid tumors [namely, adenoid cystic carcinoma (ACC)] and hematologic malignancies were eligible. CB-103 was dosed orally in cycles of 28 days at escalating doses until disease progression. Notch-activating mutations were required in a dose confirmatory cohort. Endpoints included dose-limiting toxicities (DLT), safety, tumor response, pharmacokinetics, and pharmacodynamics. Exploratory analyses focused on correlates of Notch and target gene expression.

**Results:**

Seventy-nine patients (64, 12 dose-escalation cohorts; 15, confirmatory cohort) enrolled with 54% receiving two or more lines of prior therapy. ACC was the dominant tumor type (40, 51%). Two DLTs were observed [elevated gamma-glutamyl transferase (GGT), visual change]; recommended phase II dose was declared as 500 mg twice daily (5 days on, 2 days off weekly). Grade 3–4 treatment-related adverse events occurred in 15 patients (19%), including elevated liver function tests (LFTs), anemia, and visual changes. Five (6%) discontinued drug for toxicity; with no drug-related deaths. There were no objective responses, but 37 (49%) had stable disease; including 23 of 40 (58%) patients with ACC. In the ACC cohort, median progression-free survival was 2.5 months [95% confidence interval (CI), 1.5–3.7] and median overall survival was 18.4 months (95% CI, 6.3–not reached).

**Conclusions:**

CB-103 had a manageable safety profile and biological activity but limited clinical antitumor activity as monotherapy in this first-in-human study.

**Significance:**

CB-103 is a novel oral pan-Notch inhibitor that selectively blocks the CSL–NICD interaction leading to transcriptional downregulation of oncogenic Notch pathway activation. This first-in-human dose-escalation and -confirmation study aimed to determine the safety, pharmacokinetics, and preliminary antitumor efficacy of CB-103. We observed a favorable safety profile with good tolerability and biological activity but limited clinical single-agent antitumor activity. Some disease stabilization was observed among an aggressive *NOTCH*-mutant ACC type-I subgroup where prognosis is poor and therapies are critically needed. Peripheral downregulation of select Notch target gene levels was observed with escalating doses. Future studies exploring CB-103 should enrich for patients with *NOTCH*-mutant ACC and investigate rational combinatorial approaches in tumors where there is limited success with investigational or approved drugs.

## Introduction

Notch signaling plays a critical role in many cellular processes during development to promote cell-cell communication, whereas dysregulation leads to sustained cell proliferation and potential for invasion or metastasis—the hallmarks of cancer. Signaling of the pathway occurs when ligands bind one of four Notch receptors (1–4), leading to two proteolytic cleavage steps ending with γ-secretase liberating the Notch intracellular domain (NICD) to permit nuclear translocation and binding to the Notch-specific transcription factors (1). Transcriptional activation targets downstream genes such as *NFκB, MYC*, and *BCL2* (2).

The oncogenic role of Notch signaling was first observed in T-cell acute lymphoblastic leukemia/lymphoma (T-ALL/LBL) (3) with gain-of-function or activating mutations identified in more than half of these patients (4); while subsequent studies suggest a role for aberrant Notch activation across many solid tumors (5). For example, in adenoid cystic carcinoma (ACC), a relatively uncommon salivary gland cancer with a propensity for distant or metastatic spread and lack of therapeutic options, *NOTCH1*-activating mutations occur in up to a quarter of patients (6) and identify a subgroup with more aggressive disease (7).

Given the importance of Notch signaling in human cancer, several therapeutic approaches have been investigated to inhibit pathway activation, including mAbs against Notch receptors and small-molecule γ-secretase inhibitors (GSI; ref. 8). However, constitutive downstream activation of the Notch pathway could limit signal inhibition via these approaches, and gastrointestinal tract toxicity impacts tolerability of GSIs. CB-103 is an orally bioavailable pan-Notch inhibitor that selectively blocks the CSL (CBF1, Suppressor of Hairless, Lag-1) protein–NICD interaction leading to transcriptional downregulation of oncogenic pathway activation. CB-103 therefore has the potential to address downstream mechanisms of Notch pathway signaling and overcome dose-limiting toxicities (DLT) associated with previous Notch targeting agents (9). Furthermore, CB-103 has shown potent anticancer activity as a single agent and in combination with targeted and cytotoxic therapies in preclinical models (9).

Here we present the results of the first-in-human, dose-escalation and -confirmation trial of CB-103 across multiple solid and hematologic cancer types to primarily assess safety and tolerability, pharmacokinetics, and evaluate preliminary antitumor efficacy.

## Materials and Methods

### Study Population

The study enrolled adults with histologically confirmed, locally advanced and/or metastatic solid tumors who had progressed on at least one line of prior systemic therapy (except for ACC) and relapsed/refractory T-ALL/LBL for whom no standard therapy was available. In dose escalation, participants with solid tumors with known or frequent Notch pathway–activating mutations were eligible (breast cancer, gastrointestinal tumors, hepatocellular carcinoma, osteosarcoma, malignant glomus tumor, and ACC), while the confirmatory cohort planned to enroll participants with selected tumor types (including T-ALL/LBL) and confirmed Notch pathway activation. Key eligibility criteria included patients ≥18 years of age with evaluable disease, Eastern Cooperative Oncology Group (ECOG) performance status 0–1, able to swallow capsules, and adequate organ function. Participants were excluded if they had clinically significant cardiac disease or thromboembolic events within the preceding 6 months, and drugs prolonging the QTc were avoided. A complete list of eligibility criteria is outlined in the Supplementary Materials and Methods.

### Study Design

The study was an open-label, nonrandomized, phase I/II dose-escalation study with planned expansion cohorts. In Part A, participants received CB-103 orally on a once daily schedule (28-day cycle length) which could be adapted during escalation to twice daily or intermittent dosing based on pharmacokinetic and safety signals, to determine the MTD or recommended phase II dose (RP2D). Part B was a potential expansion phase at the MTD/RP2D to determine preliminary evidence of antitumor activity and to confirm safety among patients stratified by preselected cancer indications.

Part A was based on a two-parameter Bayesian logistic regression model (BLRM) to investigate safety and tolerability of sequentially enrolled dose cohorts of 3–6 patients. The first 2 participants of each cohort were enrolled in a staggered approach with at least 1 day apart between first dosing. Each dose cohort had to complete a DLT assessment period (one treatment cycle, or 28-days for at least 3 patients) and be reviewed by a Cohort Review Committee before opening a subsequent dose cohort. DLT was defined as any grade 3–4 adverse event (AE) or abnormal lab value [according to NCI Common Terminology Criteria for Adverse Events (CTCAE) v4.03] assessed as unrelated to disease progression, intercurrent illness, or concomitant medications that occurred ≤28 days following the first dose of CB-103.

Following a 28-day screening period, a starting dose of 13 mg once daily CB-103 was based on nonclinical toxicology and biochemical studies (Supplementary Data) and expected to achieve an exposure area under the curve (AUC_0–24_) in humans of approximately 600 μg*hour/mL. This proposed starting dose was below 1/10th of the exposure of the highest nonseverely toxic dose observed in rats and dogs. Subsequent doses and schedules across 12 planned escalation cohorts were determined according to observed pharmacokinetic profile and safety.

Safety was assessed via monitoring of DLTs during the first treatment cycle, and of AEs, physical exam, and clinical lab results (hematology, coagulation, blood chemistry, urinalysis, and cardiac markers) throughout the study (Supplementary Table S1). 12-lead electrocardiograms and Holter safety monitoring with periodic cardiac imaging was employed. Efficacy assessments used RECIST v1.1 applied to CT/MRI scans performed at screening and every 8 weeks; or evaluated complete remission with complete hematologic recovery by National Comprehensive Cancer Network guideline criteria for T-ALL/T-LBL. Adherence to study drug administration was evaluated using both patient diaries and capsule counts.

### Study Endpoints

The primary endpoint for dose escalation was the number of patients experiencing DLT during the first 28-day cycle of CB-103 and for the confirmatory phase, the incidence rate, severity, and relationship of AEs to CB-103. Secondary endpoints included assessment of clinical benefit rate [CBR; defined as achieving complete and/or partial response and/or stable disease (SD) at prespecified benchmarks], duration of response, progression-free survival (PFS) and overall survival (OS), and plasma pharmacokinetics.

### Pharmacokinetics

Plasma pharmacokinetics were assessed for CB-103 via blood samples taken on days 1 (predose, 0.5, 1, 2, 4, 6, 8 hours postdose ± 5 minutes, and 12 hours postdose ± 15 minutes), 2 (24-hour post-day 1, predose), 3 (predose), 8 (predose, 0.5, 1, 2, 4, 6, and 8 hour postdose ± 5 minutes), 9 (24-hour post-day 8, predose), 15, and 22 (predose, and 1 hour postdose) in cycle 1. In cycle 2, on day 1 (predose, 0.5, 1, 2, 6, and, 8 hours postdose ± 5 minutes) and 15 (predose); in cycles 3–6 once at each visit (predose or postdose). For a twice daily dose schedule, pharmacokinetic timepoints were adjusted to evaluate concentration-versus-time curves after morning and evening doses on days 1 and 8.

CB-103 concentrations were determined by validated high-performance LC/MS-MS method and analyzed descriptively using Phoenix WinNonlin v6.3 (Pharsight Corporation). Data at timepoints collected were used to generate a population pharmacokinetic model to estimate AUC and C_max_ to associate with safety and efficacy.

### Pharmacodynamics and Biomarker Assessments

Archival tumor tissue (not older than 6 months prior to screening) or fresh tumor biopsy was required to characterize Notch alteration status by targeted genomic sequencing and/or NICD1 expression (via IHC) among the confirmatory cohort at baseline. Repeat biopsy on cycle 2 day 15 and at disease progression was optional. Serial whole blood samples (and/or bone marrow samples and saliva among patients with T-ALL/T-LBL) were obtained on day 1 of cycles 1–6 to evaluate the dynamics of Notch gene expression. Peripheral blood was collected with PaxGene tubes and frozen at −20°C until processing. RNA was extracted (Qiagen RNeasy Micro kit) from peripheral whole blood and analyzed by Nanostring with percent change in expression levels reported with respect to baseline normalized values.

### Statistical Analysis

The safety set (SS) consisted of all patients who received at least one dose of CB-103 and who had at least one postbaseline safety assessment. The SS was the primary population for all safety and efficacy analyses, except for determination of dose-DLT relationship. The dose-determining set was used to determinate the MTD and included all patients in the SS who had experienced a DLT at any time during cycle 1 and/or met the minimum requirements in cycle 1 (CB-103 dosed for ≥21 days, observed for ≥28-days after day 1, and completed cycle 1 safety evaluations).

The objective of the BLRM design (10) was to determine the MTD defined as the highest dose with less than 25% risk of the true DLT rate being above 33%. The MTD was considered reached if one of the following criteria was fulfilled: (i) if the posterior probability of the true DLT rate in the target interval (16%–33%) of the MTD was >50%, and (ii) at least 6 patients were treated at the MTD. Under this model, and accounting for dropouts and some additional patients enrolled to each cohort, approximately 55 patients were anticipated to enroll in dose escalation and approximately 45 patients in the confirmatory phase. Data were reported using 95% confidence intervals (CI) and survival endpoints (PFS, OS) are reported from the time of first treatment to the first of progression or death summarized by Kaplan–Meier estimates. All statistical analyses were analyzed using SAS v9.4 software.

### Study Oversight and Data Availability

The study was performed in accordance with the Declaration of Helsinki statement on ethical biomedical research and with the International Conference on Harmonization Guidelines for Good Clinical Practice. The study was approved by the local Institutional Review Boards for each study site. All patients provided written informed consent. The trial is registered at ClinicalTrials.gov (NCT03422679) and the full protocol is provided in the Supplementary Materials and Methods. The data generated in the study are available within the article and from the corresponding author upon request.

## Results

### Patient Characteristics

From December 2017 to January 2022, a total of 79 patients enrolled to the study, including 64 subjects to 12 escalating dose cohorts (ranging from 13 mg once daily to 500 mg twice daily 5 days on and 2 days off each week), and 15 subjects to a single confirmatory cohort dosed at 500 mg once daily (Supplementary Fig. S1). Because of limited single-agent activity at the RP2D, the study was terminated (Part A). The median age of the study cohort was 56 (range: 23–76), comprised of a mix of men and women (57% vs. 43%, respectively), with 54% having received two or more prior lines (median: 3; range: 1–7) of systemic therapy for their cancer (Table 1). Twenty-five patients (32%) had received prior radiotherapy, and most (82%) had prior cancer-related surgery. Incurable recurrent or metastatic ACC was the dominant tumor subtype (40, 51%).

**TABLE 1:** Patient demographics

	All, *n* (%) (*n* = 79)	Dose escalation cohorts^a^, *n* (%) (*n* = 64)	ACC confirmatory cohort, *n* (%) (*n* = 15)	All patients with ACC, *n* (%) (*n* = 40)^b^
Median age (y, range) <65 ≥65	56.0 (23–76) 55 (70) 24 (30)	54.8 (25–76) 43 (67) 21 (33)	43.0 (23–71) 12 (80) 3 (20)	48.5 (23–74) 31 (78) 9 (23)
Gender Male Female	45 (57) 34 (43)	35 (55) 29 (45)	10 (67) 5 (33)	21 (53) 19 (48)
Race/Ethnicity White Asian Black Other	67 (85) 10 (13) 1 (1) 1 (1)	61 (95) 1 (1) 1 (1) 1 (1)	6 (40) 9 (60) 0 0	31 (78) 8 (20) 0 1 (3)
ECOG performance status 0 1	38 (48) 41 (52)	37 (58) 27 (42)	2 (13) 13 (87)	19 (48) 21 (53)
Cancer type ACC Acinic cell carcinoma Breast Cancer Cholangiocarcinoma Colorectal cancer Osteosarcoma Prostate cancer Undifferentiated pleomorphic sarcoma T-cell ALL/LBL	40 (51) 1 (1) 6 (8) 1 (1) 22 (28) 3 (4) 2 (3) 1 (1) 3 (4)	28 (44) 1 (2) 6 (9) 1 (2) 22 (34) 3 (5) 2 (3) 1 (2) 0	12 (80) 0 0 0 0 0 0 0 3 (20)	40 (100) — — — — — — — —
Lines of prior systemic therapy 1 line (L) 2L+	43 (54) 36 (46)	26 (41) 38 (59)	9 (60) 6 (40)	21 (53) 19 (48)
Notch positive^c^	43 (54)	28 (44)	15 (100)	27 (68)

Abbreviations: ACC = adenoid cystic carcinoma, ALL = acute lymphoblastic leukemia, LBL = lymphoblastic lymphoma, ECOG = Eastern Cooperative Oncology Group, y = years.

^a^Across 12 dose-escalation cohorts (13 to 522 mg daily, and 250–500 mg twice daily).

^b^ACC subgroup is included in the *n* = 79 total study population.

^c^Notch alteration status as determined by tumor molecular profiling and verified by next-generation sequencing.

### Safety and Tolerability

Two DLTs were observed across 12 dose-escalation cohorts (*n* = 64). In cohort 8 (552 mg daily dose), 1 patient developed asymptomatic grade 3 elevation in gamma-glutamyl transferase leading to drug discontinuation, and another in cohort 11 (400 mg twice daily dosing, 5 days on and 2 off each week) developed grade 3 visual impairment leading to dose interruption, and later continued treatment at a reduced dose. The MTD was not reached (NR). The RP2D of CB-103 was declared 500 mg twice daily with a 5 day on and 2 day off weekly schedule. The decision to declare 500 mg twice daily 5 days on and 2 days off weekly as the RP2D was based on the saturation in exposure observed at this dose level, and evidence of select NOTCH target gene downregulation, which would not biologically support further escalating the dose.

Among the SS population (*n* = 79), 26 grade 3 or 4 treatment-related AEs (TRAE) were reported among 15 (19%) patients (Table 2). No deaths related to study treatment were observed. The most common grade 3–4 TRAEs were anemia (*n* = 7 events), elevated liver function tests (*n* = 5 events), and elevated serum amylase or lipase (*n* = 3 events). A total of 32 patients (41%) interrupted or required dose adjustment of CB-103 during the study, including seven (47%) in the confirmatory cohort. CB-103 was interrupted for TRAEs for a median of 5 days (range: 1–14) and most often (81% of the time) resumed at the dose prior to interruption. The primary reason for treatment discontinuation was progression of disease among 58 (73%), while 5 (6%) discontinued for toxicity (including one for DLT), and 9 (11%) withdrew.

**TABLE 2:** TRAEs reported per-patient with incidence ≥10%

	Any grade *N* (%)	Grade 1–2 *N* (%)	Grade 3 *N* (%)	Grade 4 *N* (%)
Any AE	60 (76)	47 (60)	13 (16)	2 (3)
Gastrointestinal disorders	39 (49)	38 (48)	1 (1)	—
Nausea	17 (22)	17 (22)	—	—
Diarrhea	10 (13)	10 (13)	—	—
Dyspepsia	10 (13)	10 (13)	—	—
Vomiting	9 (11)	8 (10)	1 (1)	—
Eye disorders	37 (47)	36 (46)	1 (1)	—
Dyschromatopsia	15 (19)	15 (19)	—	—
Vision blurred	12 (15)	12 (15)	—	—
Visual impairment	8 (10)	7 (9)	1 (1)	—
Blood and lymphatic system disorders	13 (17)	9 (11)	4 (5)	—
Anemia	13 (16)	9 (11)	4 (5)	—
General disorders and administration site conditions	13 (16)	9 (11)	1 (1)	—
Fatigue	9 (11)	9 (11)	—	—
Laboratory investigations	13 (16)	8 (10)	5 (6)	2^a^ (3)
Skin and subcutaneous tissue disorders	8 (10)	6 (8)	2 (3)	—

NOTE: *N* = 79 patients included. Grading of adverse events per NCI Common Terminology Criteria for Adverse Events [CTCAE] v4.03.

^a^The two TRAEs classified as grade 4 were elevated lipase (one event) and elevated liver function tests (one event).

### Efficacy and Survival

**FIGURE 1:**
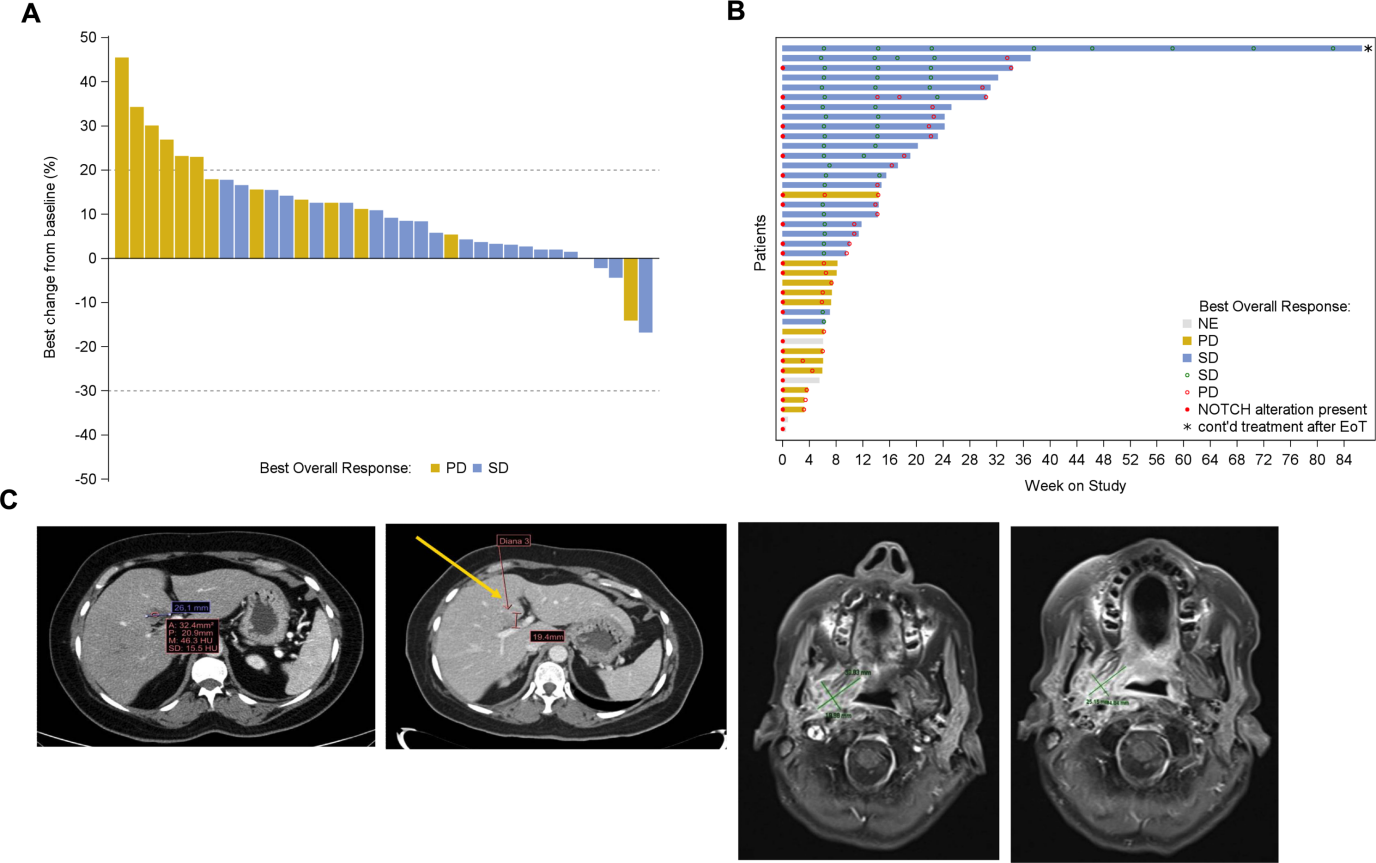
Waterfall (**A**) and swimmer (**B**) plot among all *n* = 40 patients with evaluable ACC color coded by best overall response. Time to progression is denoted along with patient survival and Notch signaling alteration status at last data cutoff (March 5, 2022). **C,** Case example of a patient with metastatic ACC with rapid progression prior to study entry showing −25.7% tumor regression in her dominant liver lesion (26.1–19.4 mm from left to right, top) and stable disease over 8 months on study; and a patient with locoregionally recurrent ACC (*NOTCH*-activating mutation present) with −17% tumor regression in a right parapharyngeal mass (30 to 25 mm from left to right, bottom) with stable disease over 3 months while on study.

Among all 76 solid tumor patients (including the confirmatory cohort and all patients with ACC), there were 37 (49%) demonstrating SD but no objective responses (Table 3; Fig. 1). The 3- and 6-month CBR was 48.7% (95% CI, 37.0–60.4) and 15.8% (95% CI, 8.4–26.0), respectively, across the study. Among the ACC cohort (*n* = 40), 23 (58%) exhibited SD with a 3- and 6-month CBR of 57.5% (95% CI, 40.9–73.0) and 27.5% (95% CI, 14.6–43.9), respectively (Table 3).

**TABLE 3:** Efficacy and survival outcomes for patients with solid tumor

	All patients (*n* = 76)^a^	ACC confirmatory cohort (*n* = 12)^a^	All patients with ACC (*n* = 40)
Best overall response (*n*, %) Complete response Partial response Stable disease Progressive disease Unevaluable	0 0 37 (49) 29 (38) 10 (13)	0 0 3 (25) 7 (58) 2 (17)	0 0 23 (58) 13 (33) 4 (10)
Clinical benefit rate (%, 95% CI) 3 months 6 months 9 months	48.7 (37.0–60.4) 15.8 (8.4–26.0) 1.3 (0.0–7.1)	25.0 (5.5–57.2) — —	57.5 (40.9–73.0) 27.5 (14.6–43.9) 2.5 (0.1–13.2)
Progression-free survival No. of events (*n*, %) Median PFS (months, 95% CI) 3-month PFS 6-month PFS 9-month PFS 12-month PFS	59 (78) 1.9 (1.4–3.2) 38.7 (26.5–50.6) 15.5 (7.5–26.0) 5.8 (1.7–14.3) 3.9 (0.7–11.6)	10 (83) 1.4 (0.8–2.3) 10.9 (0.6–38.0) — — —	35 (88) 2.5 (1.5–3.7) 46.8 (30.4–61.7) 18.6 (7.9–32.8) 6.2 (1.1–17.8) 6.2 (1.1–17.8)
Overall survival No. of deaths (*n*, %) Median OS (months, 95% CI) 6-month OS 12-month OS	33 (43) 9.2 (6.3–18.4) 68.4 (54.4–78.9) 41.6 (28.0–54.7)	4 (33) 5.4 (1.3–5.4) — —	15 (38) 18.4 (8.3–NR) 72.4 (53.1–84.8) 57.3 (37.5–73.0)
Median follow-up time (months)	5.4	1.8	5.9

Abbreviations: ACC = adenoid cystic carcinoma, CI = confidence interval, NR = not reached, PFS = progression-free survival, OS = overall survival.

^a^ *n* = 3 patients with hematologic malignancies were excluded from above.

**FIGURE 2:**
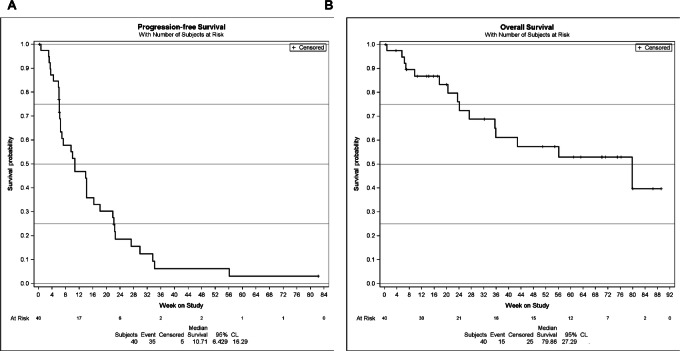
PFS (**A**) and OS (**B**) among the ACC subgroup (*n* = 40). Kaplan–Meier method; number of events (%), and median and 95% CIs shown.

At a median follow-up of 5.4 months (range: 0.1–20.5+), median PFS for the entire solid tumor cohort was 1.9 months (95% CI, 1.4–3.2) with a 6-month PFS estimate of 15.5% (95% CI, 7.5–26.0), which was similar across dose-escalation and confirmatory dose cohorts. Among the ACC cohort, median PFS was 2.5 months (95% CI, 1.5–3.7) with a 6-month PFS estimate of 18.6% (95% CI, 7.9–32.8; Table 3). Median OS for the entire solid tumor cohort was 9.2 months (95% CI, 6.3–18.4) with 33 (43%) events observed, yielding a 6- and 12-month OS rate of 68.4% and 41.6%, respectively. In the ACC subgroup, median OS was 18.4 months (95% CI, 6.3–NR) with a 6- and 12-month OS rate of 72.4% and 57.3%, respectively (Fig. 2).

### Pharmacokinetics

Robust pharmacokinetic sampling was performed across 7 days in cycle 1, 2 days in cycle 2, and before and after CB-103 dosing in cycles 3–6. Mean pharmacokinetic plasma concentrations (ng/mL) increased across dose-escalation cohorts 1 through 8 (522 mg once daily) demonstrating a peak (t_max_) around 0.5–2 hours on day 1 of cycle 1 (Supplementary Table S2; Supplementary Fig. S2). Plasma elimination half-life (t1/2) was 16.5 hours (SD, 8.5) on cycle 1 day 1 and peaked at 37.7 hours (SD, 42.6) by cycle 1 day 8. Mean AUC from 0 to 24 (hour*ng/mL) was 3155.3 (SD, 3989.4) across the entire sampled cohort with a mean C_max_ (ng/mL) on cycle 1 day 1 of 730.8 (SD, 869.8) which increased by cycle 1 day 8.

### Exploratory Biomarkers

Among the study cohort (*n* = 79), Notch signaling activation in tumor was confirmed in 43 (54%) patients, including among all 15 patients treated in the confirmatory ACC cohort [and 28/64 (44%) patients across the dose-escalation cohorts which belonged to the following tumor types: 16 ACC, eight mCRC, two mTNBC, one pleosarcoma, and one cholangiocarcinoma]. Patients with *NOTCH*-altered ACC were included across all dose ranges. Among patients with ACC, clinical benefit at 3 months was observed in 6/12 (50%) with an activating *NOTCH* mutation (Supplementary Fig. S3). When estimating total disease burden (as the sum of diameters of all target lesions in millimeters per RECIST v1.1), those patients with ACC with lower median values had a trend toward an improved 3-month clinical benefit (*P* = 0.07; Mann–Whitney test) regardless of Notch status or dose level. Furthermore, the presence of bone metastases was associated with less clinical benefit at 3 months (*P* = 0.02; Fisher exact test). One patient (64-year-old woman) with *NOTCH1*-activated metastatic ACC and tumor BCL2 overexpression had SD to CB-103 (250 mg twice daily) for 3 months, and upon signs of disease progression off-label Venetoclax (BCL2 inhibitor) was added to CB-103 resulting in further disease stabilization for an additional 3.5 months, with good tolerance in combination. Circulating tumor DNA demonstrated alterations in *TP53* and the PI3K pathway at second progression on the combination.

Percent change (%) in peripheral blood cell Notch target gene expression was monitored among a subset of patients with ACC receiving twice daily dosing (*n* = 13). Some peripherally measured Notch target genes (*HEY1, IL7R alpha, NOTCH1, 2* and *4*) expression levels declined with increasing exposure to CB-103 in plasma, up to a dose of 1,000 mg (500 mg twice daily; Supplementary Table S3).

## Discussion

This phase I/II study of the novel, oral pan-Notch inhibitor CB-103 determined the RP2D as 500 mg twice daily utilizing a 5 day on and 2 day off weekly schedule. Overall, CB-103 was well tolerated with 15 of 79 patients (19%) experiencing grade 3 or 4 AEs which were all reversible, with no deaths related to study drug, and only 5 patients (6%) discontinuing study drug for toxicity. In addition, CB-103 demonstrated favorable pharmacokinetic properties with mean plasma concentrations increasing across dose escalation.

Among all 76 evaluable solid tumor patients (most with ACC) there were no objective responses observed, but 58% of ACC patients exhibited SD with a 3- and 6-month CBR of 58% and 28%, respectively. Notably, 68% (27/40) of patients with ACC in our trial had confirmed *NOTCH*-activating tumor mutations which enriched for a subpopulation with advanced stage disease, a high rate of distant metastases, and short OS (ref. 7; see Supplementary Table S4 for representativeness of study participants). Recently, the results of ACCURACY, a phase II trial of the GSI AL101 were reported in patients with known *NOTCH*-mutated advanced ACC (11). Among 77 patients, the partial response rate was 12% with 57% of patients exhibiting SD (disease control rate: 69%, benchmark not reported). Despite a lack of objective responses observed with CB-103, disease stability rates were comparable with those observed with AL101 in this aggressive ACC population. Given the mechanism of action, further enrichment by Notch status may yield objective antitumor activity. We did observe that patients with ACC in the current study with lower tumor burden and without bone metastases had a trend toward disease stabilization. However, our trial enrolled heavily pretreated patients with ACC often with distant metastases outside the lungs (liver = 24, 60%; bone = 14, 35%). It is worth noting that CB-103 is a transcriptional modifier which might have a longer time to effect when compared with cytotoxic agents.

GSIs can result in diarrhea and fatigue in >60% of patients but grade 3+ AE rates were reportedly low. Gastrointestinal toxicities associated with GSIs were not observed with CB-103. CB-103 has an independent mechanism from GSIs, resulting in a distinct AE profile (anemia, visual changes) which also appears manageable. This is important to highlight as the broader advanced ACC population (regardless of Notch status) is often offered treatment with oral VEGFR tyrosine kinase inhibitors (TKI) such as lenvatinib and axitinib which have reported discontinuation rates of 20% or greater for toxicity (12–14). The safety profiles of CB-103 make it feasible to combine with kinase inhibitors or TKIs in future trials to augment efficacy or promote synergism.

Ferrarotto and colleagues have characterized the poor prognosis and aggressive disease phenotype that distinguishes *NOTCH1*-mutant ACC from a more indolent ACC subtype (7). Median OS for the *NOTCH1*-mutant ACC subgroup (*n* = 14) in their report was 30 months, whereas we observed a shorter median OS under 2 years in our heavily pretreated cohort. Their more recent work has clarified the *NOTCH*-mutant cases as part of an ACC-I subclass that collectively demonstrate upregulation of other hyperactivating genes such as *MYC* and *BCL2*, which describes an estimated 37% of all ACC tumors (15). ACC-I is also associated with solid component histology and minor salivary tissue origin. Mechanistically, Notch signaling activation is thought to drive MYC transcriptional upregulation and overexpression (4). Of interest, resistance to GSIs has been linked with alternative MYC expression via Notch-independent signaling (16). While CB-103 inhibits the CSL protein–NICD interaction aiming to facilitate transcriptional downregulation, alternative enhancers may be a mechanism of acquired resistance.

Another potential target in ACC-I is the apoptotic protein *BCL2*, and again upregulation of the gene with *MYC* and NICD1 coexpression has been demonstrated (14). Preclinical data from myeloma cell lines have suggested synergism when combining a GSI with a novel BH3 mimetic (ABT-737) known to block BCL2/BCLXL to induce apoptosis (17). Su and colleagues have also shown that knockdown of *NOTCH1* in salivary ACC cell lines inhibits downstream BCL2 (18). Investigating Notch inhibiting agents such as CB-103 with BCL2 inhibitors represents a rationale combinatorial strategy. In the current study, 1 patient with *NOTCH1*-activating metastatic ACC and tumor BCL2 overexpression had additional tumor growth stabilization with the off-label addition of the BCL2 inhibitor Venetoclax to CB-103 after first progression.

In conclusion, the novel oral pan-NOTCH inhibitor CB-103 demonstrated a manageable safety profile with good tolerability and biological activity despite limited antitumor activity as monotherapy in this first-in-human study. We observed some evidence of disease stabilization in an aggressive ACC-I type population where prognosis is poor, and therapies are urgently needed. Further enrichment for *NOTCH*-mutant ACC and rationale combinatorial approaches that aim to address mechanisms of Notch signaling resistance would be important in future studies exploring CB-103.

## Supplementary Material

Supplementary Figure 1 Study design, dose escalation, and confirmatory dosing cohorts (Part A) for CB-103

Supplementary Figure 2 Pharmacokinetic parameters

Supplementary Figure 3 Biomarkers of Clinical Benefit for ACC patients

Supplementary Table 1 Safety and toxicity monitoring throughout the CB-103 clinical tria1

Supplementary Table 2 Pharmacokinetic parameters for CB-103

Supplementary Table 3 Pharmacodynamic and biomarker assessments: percent change in peripheral Notch target gene expression in circulation across individual patients (each row) on twice daily dosing of CB-103

Supplementary Table 4 Representativeness of Study Participants
