# Supplementary material for: A Phase I Study of the Pan-Notch Inhibitor CB-103 for Patients with Advanced Adenoid Cystic Carcinoma and Other Tumors
Source: Cancer Res Commun. 2023 Sep 14;3(9):1853–61. doi: 10.1158/2767-9764.CRC-23-0333 (PMC10501326; doi:10.1158/2767-9764.CRC-23-0333)
Supplement: Supplementary Figure 1 — Study design, dose escalation, and confirmatory dosing cohorts (Part A) for CB-103 [file crc-23-0333-s01.pdf]

Supplemental Figure 1. Study design, dose escalation, and confirmatory dosing cohorts (Part A) for CB-103

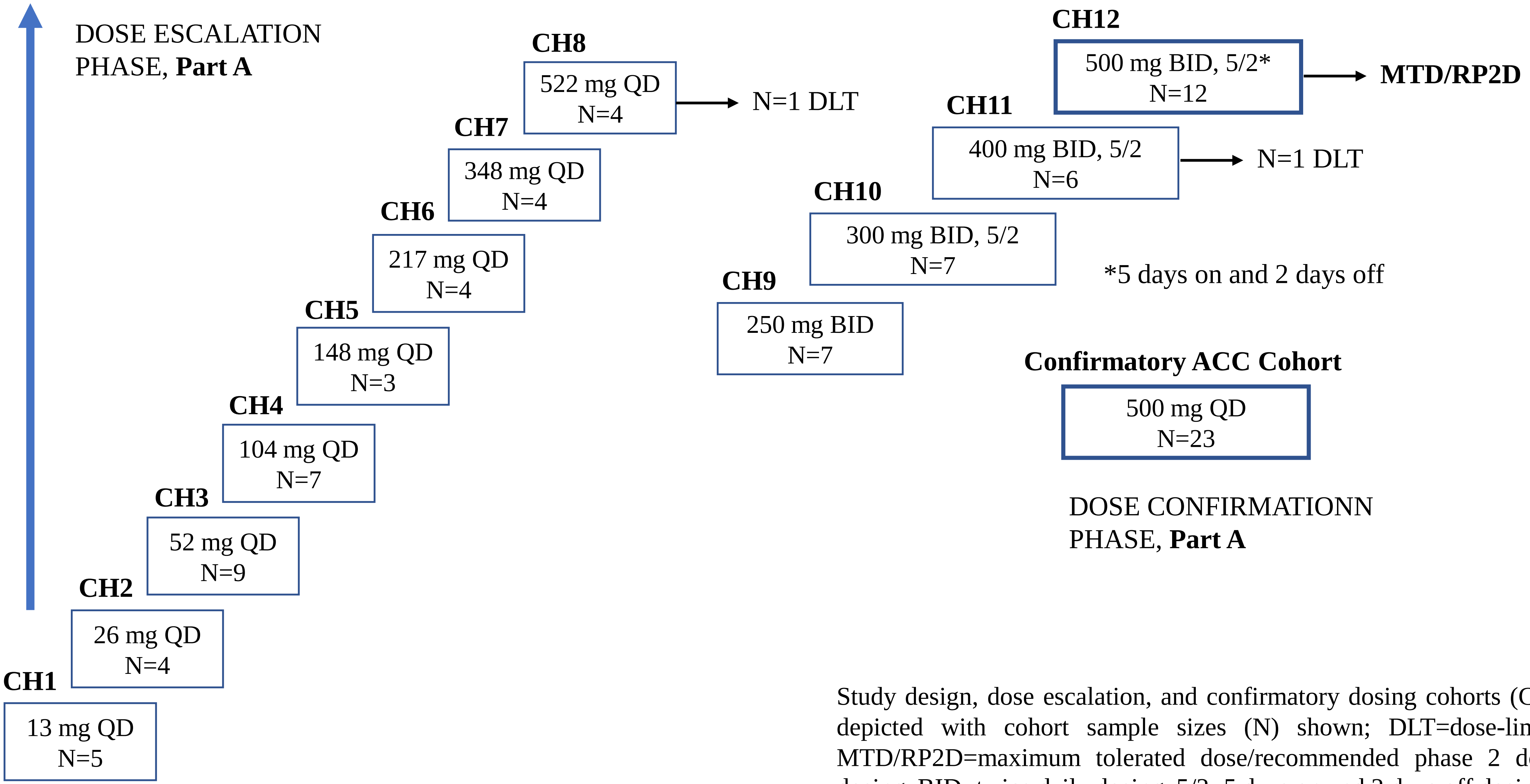

Study design, dose escalation, and confirmatory dosing cohorts (CH) graphically depicted with cohort sample sizes (N) shown; DLT=dose-limiting toxicity. MTD/RP2D=maximum tolerated dose/recommended phase 2 dose. QD=daily dosing. BID=twice daily dosing. 5/2=5 days on and 2 days off dosing.

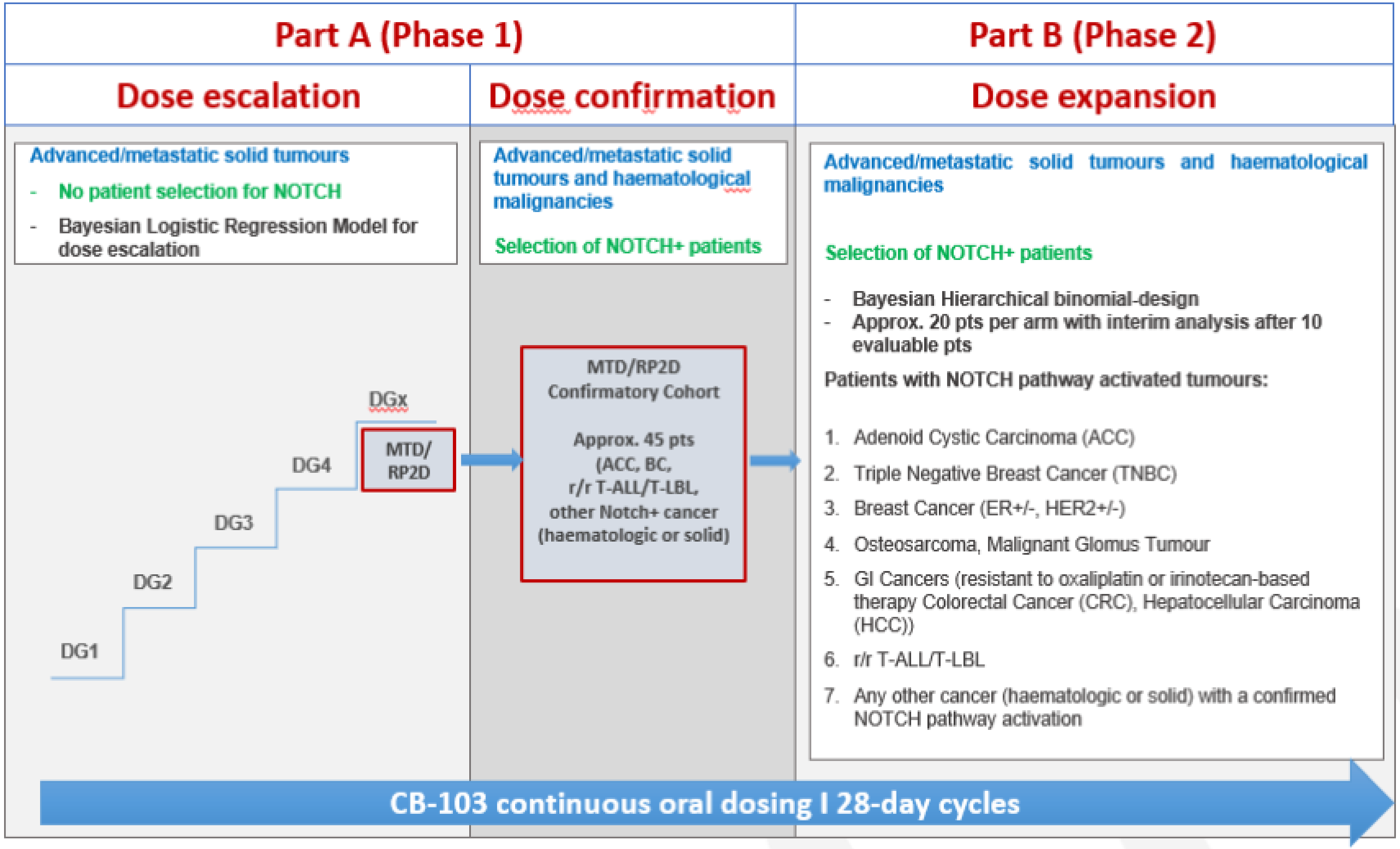

Abbreviations: ACC, adenoid cystic carcinoma; DG, dose group; MTD, maximum tolerated dose; pts, patients; ER, estrogen receptor; CRC, colorectal cancer; HCC, hepatocellular carcinoma; HER2, human epidermal growth factor receptor 2; PR, progesterone receptor; r/r, relapsed/refractory; RP2D, recommended phase 2 dose; TNBC, triple negative breast cancer.
