# Supplementary material for: A Phase I Study of the Pan-Notch Inhibitor CB-103 for Patients with Advanced Adenoid Cystic Carcinoma and Other Tumors
Source: Cancer Res Commun. 2023 Sep 14;3(9):1853–61. doi: 10.1158/2767-9764.CRC-23-0333 (PMC10501326; doi:10.1158/2767-9764.CRC-23-0333)
Supplement: Supplementary Figure 2 — Pharmacokinetic parameters [file crc-23-0333-s02.pdf]

Supplemental Figure 2. Pharmacokinetic parameters

A **B**

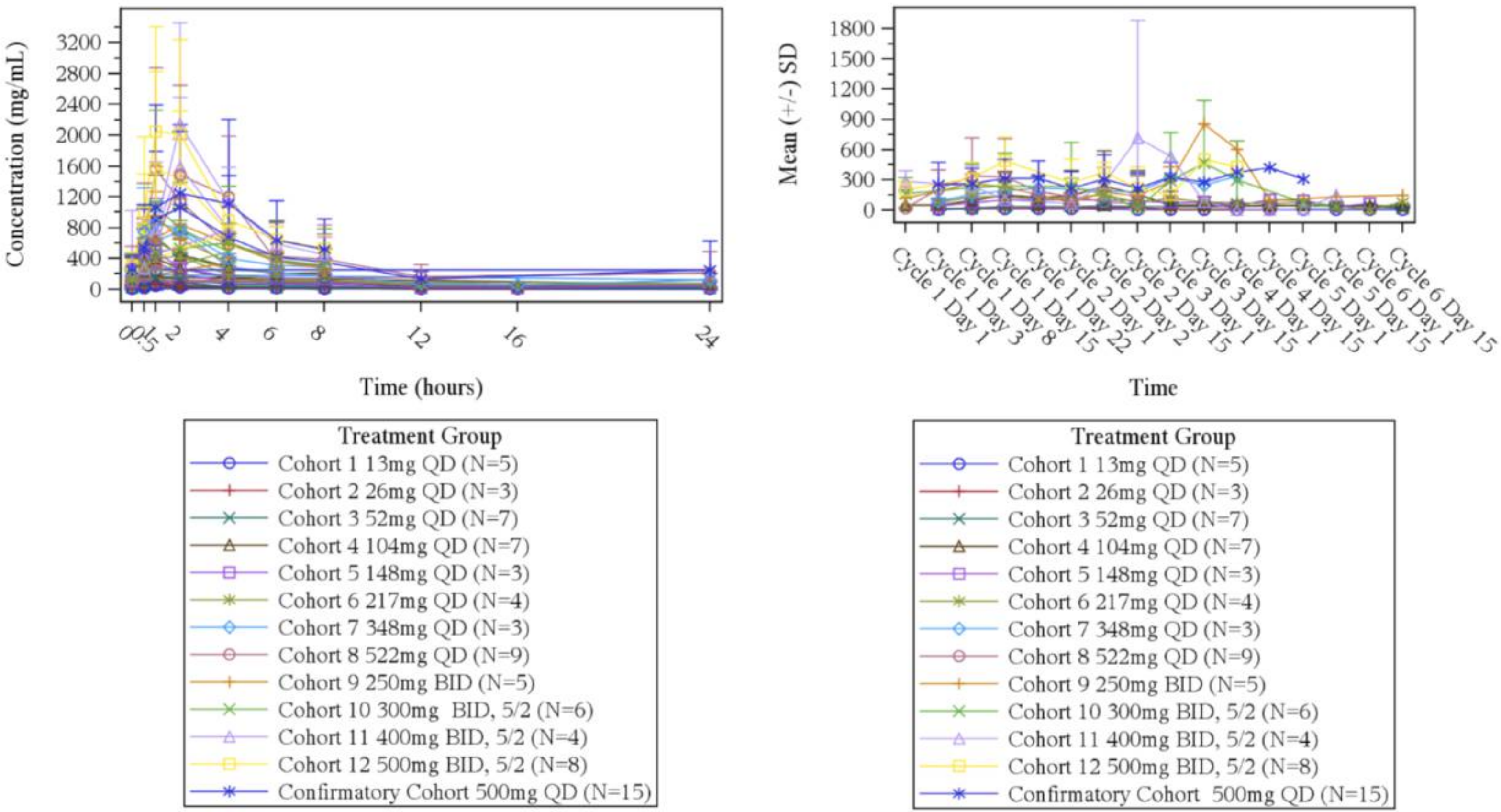

(A) Plasma drug concentrations (ng/mL) of CB-103 measured at pre-specified timepoints over 24 hours on cycle 1 day 1 of dosing. **(B)** Pre-dose mean plasma drug concentration (ng/mL) of CB-103 measured through cycle 6 of treatment. Mean Standard deviation denoted by error bars; color coding by dose escalation and confirmatory dose cohort subgroups.
