# Supplementary material for: A Phase I Study of the Pan-Notch Inhibitor CB-103 for Patients with Advanced Adenoid Cystic Carcinoma and Other Tumors
Source: Cancer Res Commun. 2023 Sep 14;3(9):1853–61. doi: 10.1158/2767-9764.CRC-23-0333 (PMC10501326; doi:10.1158/2767-9764.CRC-23-0333)
Supplement: Supplementary Figure 3 — Biomarkers of Clinical Benefit for ACC patients [file crc-23-0333-s03.pdf]

Supplemental Figure 3. Biomarkers of Clinical Benefit for ACC patients

A

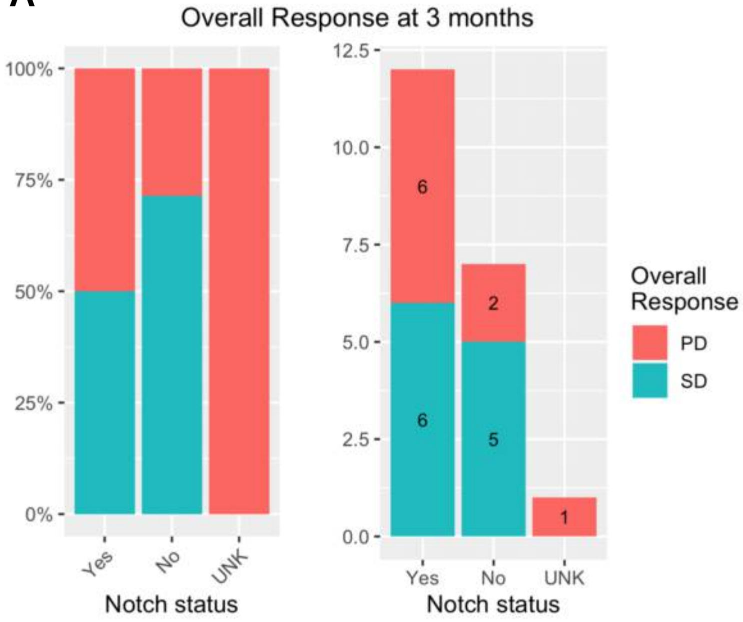

B

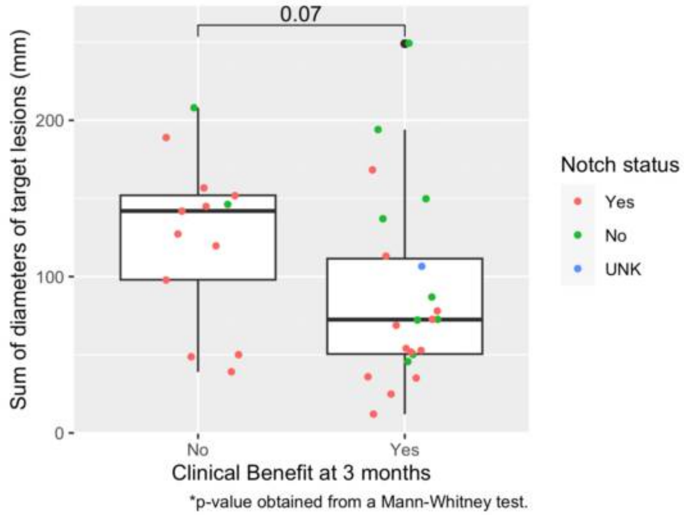

C

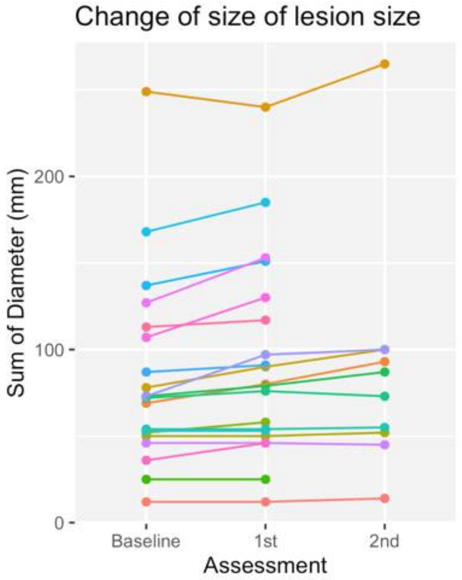

D

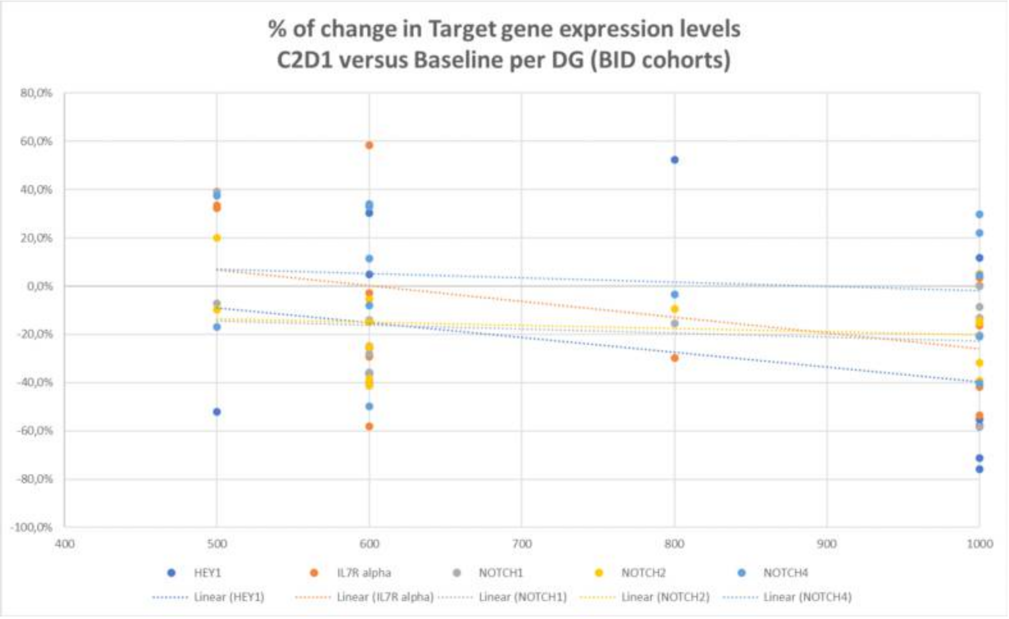

(A) Histograms showing overall response at 3 months color-coded by stable disease (SD) or progressive disease (PD) and plotted as a function of Notch mutational status (yes=positive, no=negative, unk=unknown). **(B)** Box and whisker plot showing clinical benefit or SD rate at 3-month benchmark (Y=yes or N=no) plotted against total tumor burden (sum of diameters of all target lesions in millimeters [mm]) and color-coded by Notch mutational status. **(C)** Dot plot showing individual baseline or pre-treatment patient tumor burden (sum of diameter of target lesions in millimeters) over time to 1st assessment at 6-weeks and 2nd assessment at 12-weeks (according to RECIST v1.1); each patient represents a colored dot-line. **(D)** Dot plot showing percent change (%) in target Notch gene expression levels at cycle 2, day 1 compared with baseline among a range of twice daily (BID cohorts) dose groups (DGs) from left to right.
