# Supplementary material for: A Phase I Study of the Pan-Notch Inhibitor CB-103 for Patients with Advanced Adenoid Cystic Carcinoma and Other Tumors
Source: Cancer Res Commun. 2023 Sep 14;3(9):1853–61. doi: 10.1158/2767-9764.CRC-23-0333 (PMC10501326; doi:10.1158/2767-9764.CRC-23-0333)
Supplement: Supplementary Table 1 — Safety and toxicity monitoring throughout the CB-103 clinical tria1 [file crc-23-0333-s04.docx]

**Supplemental Table 1.** Safety and toxicity monitoring throughout the CB-103 clinical tria1

|  | Cycle 1 | Cycle 2 | Cycle 3 and beyond | Other |
| --- | --- | --- | --- | --- |
| Safety assessments* | Day 1, 2, 3, 8, 9, 15, 22 | Day 1, 2, 8, 15 | Day 1, 15. Only Day 1 after Cycle 6 | EOT, follow-up |
| Hematology labs | Day 1, 3, 8, 15, 22 | Day 1, 15 | Day 1, 15. Only Day 1 after Cycle 6 | EOT, follow-up |
| Clinical chemistry labs | Day 1, 3, 8, 15, 22 | Day 1, 15 | Day 1, 15. Only Day 1 after Cycle 6 | EOT, follow-up |
| Electrocardiogram (ECG)** | Day 1, 8, 15, 22 | Day 1, 15 | -- | EOT |

*at each study visit

**echocardiogram or Multigated Acquisition Scan (MUGA) performed for left ventricular function assessment, Holter monitoring, and cardiac serum markers were also assessed periodically.

EOT=end of treatment
