# Supplementary material for: A Phase I Study of the Pan-Notch Inhibitor CB-103 for Patients with Advanced Adenoid Cystic Carcinoma and Other Tumors
Source: Cancer Res Commun. 2023 Sep 14;3(9):1853–61. doi: 10.1158/2767-9764.CRC-23-0333 (PMC10501326; doi:10.1158/2767-9764.CRC-23-0333)
Supplement: Supplementary Table 2 — Pharmacokinetic parameters for CB-103 [file crc-23-0333-s05.docx]

**Supplemental Table 2.** Pharmacokinetic parameters for CB-103

|  | **Cycle 1, Day 1** | | | | **Cycle 1, Day 8** | | | |
| --- | --- | --- | --- | --- | --- | --- | --- | --- |
|  | Mean Cmax | Cmax SD (ng/mL) | Mean AUC24 (h*ng/mL) | AUC24 SD (h*ng/mL) | Mean Cmax | Cmax SD (ng/mL) | Mean AUC24 (h*ng/mL) | AUC24 SD (h*ng/mL) |
| Cohort 1 (13 mg) | 44.40 | 28.78 | 173.66 | 117.56 | 58.74 | 30.51 | 432.56 | 179.78 |
| Cohort 2 (26 mg) | 53.68 | 77.36 | 219.65 | 254.95 | 555.80 | 56.43 | 71.20 | 458.21 |
| Cohort 3 (52 mg) | 98.47 | 50.75 | 496.23 | 231.01 | 154.41 | 93.69 | 999.15 | 395.71 |
| Cohort 4 (104 mg) | 367.60 | 1899.36 | 1442.41 | 329.25 | 418.11 | 535.55 | 3688.76 | 5887.45 |
| Cohort 5 (148 mg) | 481.63 | 700.08 | 1261.85 | 1256.75 | 426.67 | 467.23 | 2825.14 | 2145.42 |
| Cohort 6 (217 mg) | 731.00 | 599.53 | 2620.13 | 1418.30 | 912.50 | 607.05 | 4249.52 | 2359.42 |
| Cohort 7 (348 mg) | 1152.00 | 390.70 | 3517.77 | 1407.40 | 873.67 | 98.88 | 6884.68 | 1022.55 |
| Cohort 8 (522 mg) | 1227.38 | 1031.57 | 5905.64 | 4655.95 | 1740.048 | 1240.91 | 13541.36 | 11495.68 |
| Cohort 9 (250 mg BID) | 1013.64 | 806.08 | 6142.81 | 5228.1 | 1305.67 | 461.18 | 11660.56 | 3248.72 |
| Cohort 10 (300 mg BID 5/2) | 1631.67 | 1740.57 | 10379.90 | 9273.23 | 1389.60 | 550.68 | 11311.09 | 6306.14 |
| Cohort 11 (400 mg BID 5/2) | 1617.50 | 377.48 | 14867.46 | 3276.11 | 2403.33 | 1210.84 | 14059.65 | 6034.43 |
| Cohort 12 (500 mg BID 5/2) | 2385.63 | 1309.34 | 14216.41 | 6170.50 | 2761.43 | 1035.59 | 18614.07 | 6400.69 |

AUC=area under the curve; SD=standard deviation; BID=twice daily dosing.
