# Supplementary material for: A Phase I Study of the Pan-Notch Inhibitor CB-103 for Patients with Advanced Adenoid Cystic Carcinoma and Other Tumors
Source: Cancer Res Commun. 2023 Sep 14;3(9):1853–61. doi: 10.1158/2767-9764.CRC-23-0333 (PMC10501326; doi:10.1158/2767-9764.CRC-23-0333)
Supplement: Supplementary Table 3 — Pharmacodynamic and biomarker assessments: percent change in peripheral Notch target gene expression in circulation across individual patients (each row) on twice daily dosing of CB-103 [file crc-23-0333-s06.docx]

**Supplemental Table 3.** Pharmacodynamic and biomarker assessments: percent change in peripheral Notch target gene expression in circulation across individual patients (each row) on twice daily dosing of CB-103

| Total dose (mg) | Cmax (h*ng/mL) | *HEY1* | *IL7R alpha* | *NOTCH1* | *NOTCH 2* | *NOTCH4* |
| --- | --- | --- | --- | --- | --- | --- |
| 500 | 1230 | -- | 32.3% | 39.2% | 19.9% | 37.6% |
| 500 | 1800 | -52.0% | 33.5% | -7.1% | -9.6% | -16.9% |
| 600 | 638 | 5.0% | 58.3% | -28.1% | -25.2% | 34.0% |
| 600 | 2100 | -36.1% | -58.0% | -41.2% | -38.4% | -49.7% |
| 600 | 1280 | -36.1% | -24.8% | -35.7% | -5.5% | -7.9% |
| 600 | 1220 | 30.3% | -2.9% | -14.2% | -14.8% | 11.5% |
| 600 | 1710 | -25.6% | -29.3% | -39.7% | -41.1% | 32.9% |
| 800 | 1730 | 52.3% | -29.7% | -15.5% | -9.4% | -3.5% |
| 1000 | 4420 | 11.7% | -53.6% | -8.7% | -31.8% | -20.8% |
| 1000 | 2580 | -71.3% | 3.5% | -0.1% | -14.2% | -40.4% |
| 1000 | 3760 | -75.9% | -16.2% | -58.3% | -39.3% | 22.0% |
| 1000 | 2410 | -55.2% | 0.2% | -20.2% | -15.2% | 4.5% |
| 1000 | 1240 | -57.8% | -41.7% | -13.3% | 5.1% | 29.7% |
