# Supplementary material for: A Phase I Study of the Pan-Notch Inhibitor CB-103 for Patients with Advanced Adenoid Cystic Carcinoma and Other Tumors
Source: Cancer Res Commun. 2023 Sep 14;3(9):1853–61. doi: 10.1158/2767-9764.CRC-23-0333 (PMC10501326; doi:10.1158/2767-9764.CRC-23-0333)
Supplement: Supplementary Table 4 — Representativeness of Study Participants [file crc-23-0333-s07.docx]

**Supplemental Table 4.** Representativeness of Study Participants

| Cancer type(s)/subtype(s)/stage(s)/condition | Adenoid cystic carcinoma (ACC) |
| --- | --- |
| Considerations related to: | |
| Sex | In a recent population study, ACC occurred more commonly in women than men (60:40 ratio). |
| Age | ACC has a preponderance for the elderly, with the highest incidence of cases of ACC in the head and neck area seen in the fifth to sixth decade of life. |
| Race/ethnicity | Owing to the rarity of these tumors, there is little data on whether specific ethnic, geographic, exposure, or other factors predispose to the development of the disease. |
| Geography | Owing to the rarity of these tumors, there is little data on whether specific ethnic, geographic, exposure, or other factors predispose to the development of the disease. |
| Other considerations | The contribution of the solid component is a factor of the aggressiveness of the tumor. A grading strategy is used to describe the histology. In ACC, *NOTCH1* mutations (occurring in 15-25% of tumors) define a distinct disease phenotype characterized by solid histology, liver and bone metastasis, poor prognosis, and potential responsiveness to Notch inhibitors. |
| Overall representativeness of this study | The sex and age distribution of our study is similar to the average age distribution of ACC in the literature. Notably, this study enriched for the more aggressive ACC subtype by including many patients with established Notch pathway alterations in their tumors. |
